# Comparison of techniques for handling missing covariate data within prognostic modelling studies: a simulation study

**DOI:** 10.1186/1471-2288-10-7

**Published:** 2010-01-19

**Authors:** Andrea Marshall, Douglas G Altman, Patrick Royston, Roger L Holder

**Affiliations:** 1 Centre for Statistics in Medicine, University of Oxford, Oxford, UK; 2 Warwick Clinical Trials Unit, University of Warwick, Coventry, UK; 3 Hub for Trials Methodology Research and UCL, MRC Clinical Trials Unit, London, UK; 4 Department of Primary Care Clinical Sciences, University of Birmingham, Birmingham, UK

## Abstract

**Background:**

There is no consensus on the most appropriate approach to handle missing covariate data within prognostic modelling studies. Therefore a simulation study was performed to assess the effects of different missing data techniques on the performance of a prognostic model.

**Methods:**

Datasets were generated to resemble the skewed distributions seen in a motivating breast cancer example. Multivariate missing data were imposed on four covariates using four different mechanisms; missing completely at random (MCAR), missing at random (MAR), missing not at random (MNAR) and a combination of all three mechanisms. Five amounts of incomplete cases from 5% to 75% were considered. Complete case analysis (CC), single imputation (SI) and five multiple imputation (MI) techniques available within the R statistical software were investigated: a) data augmentation (DA) approach assuming a multivariate normal distribution, b) DA assuming a general location model, c) regression switching imputation, d) regression switching with predictive mean matching (MICE-PMM) and e) flexible additive imputation models. A Cox proportional hazards model was fitted and appropriate estimates for the regression coefficients and model performance measures were obtained.

**Results:**

Performing a CC analysis produced unbiased regression estimates, but inflated standard errors, which affected the significance of the covariates in the model with 25% or more missingness. Using SI, underestimated the variability; resulting in poor coverage even with 10% missingness. Of the MI approaches, applying MICE-PMM produced, in general, the least biased estimates and better coverage for the incomplete covariates and better model performance for all mechanisms. However, this MI approach still produced biased regression coefficient estimates for the incomplete skewed continuous covariates when 50% or more cases had missing data imposed with a MCAR, MAR or combined mechanism. When the missingness depended on the incomplete covariates, i.e. MNAR, estimates were biased with more than 10% incomplete cases for all MI approaches.

**Conclusion:**

The results from this simulation study suggest that performing MICE-PMM may be the preferred MI approach provided that less than 50% of the cases have missing data and the missing data are not MNAR.

## Background

Assessing the prognostic ability of clinical factors using a Cox proportional hazards model is often performed [1]. However, missing covariate data complicates the analysis, but often occurs [1]. A review of published prognostic studies [1] found that on average 13% of cases had incomplete data (range 0 - 60%) in 39 studies where this information could be obtained. In addition, 27% of values, on average, were missing within a single variable (range 0 - 72%) in 55 studies [1]. Simply using the cases with complete covariate data, i.e. performing a complete case (CC) analysis, loses information and hence efficiency, and may lead to biased regression coefficients if the missingness is related to the outcome [2,3]. Sophisticated likelihood based techniques can explicitly handle missing covariate data in analyses of survival (time to event) data (e.g. [4-6]). However, these generally require problem-specific programs to be written and hence may not be readily available.

Imputing the missing data poses a suitable alternative that uses all the data and can be performed using easily accessible methods. Multiple imputation (MI), where each missing value is replaced with a set of *m *(>1) independent values [7] to give *m *separate complete datasets, incorporates uncertainty of the missing data that cannot be achieved with single imputation (*m *= 1). The *m *completed datasets are analysed individually using standard statistical methods and the results combined into one summary estimate using simple rules devised by Rubin [7]. The parameter estimates of interest are averaged and a variance estimate is obtained that incorporates both the within and between imputation variability. There are many different techniques for performing MI, but most approaches assume the missing data to be at least missing at random (MAR), where the probability of missingness is only associated with the observed and not the unobserved data [8]. MI approaches are generally based on an imputation model from which plausible values for the missing data are obtained. The imputation model should contain all variables to be subsequently analysed, which for prognostic modelling studies includes the outcome and all potential covariates, but also any variables that help to explain the missing data [9]. The more compatible the imputation and analysis models are, the more successful the MI approach will be [10]. However, the use of MI in the published medical literature remains limited [11].

Simulation studies provide a framework to compare the performance of different approaches for handling missing data with a variety of missing data mechanisms, as the true value is known. Several simulation studies have investigated the effects of missing data using different MI approaches, but these have primarily imposed missingness only on the outcome variable (e.g. [11,12]). These studies demonstrated that model based imputation approaches for an incomplete outcome variable were better than ad hoc imputation procedures and were fairly robust to some model departures [13]. Furthermore, when a fully parametric imputation model correctly fitted the data, it performed better than alternative techniques such as predictive mean matching [14]. Conversely, fully parametric methods performed worse when the imputation model did not fit [15]. Few simulation studies have considered missing covariate data (e.g. [3]), especially situations where missingness was imposed on more than one covariate (e.g. [11,16]). Only a limited number of these studies included survival as the outcome and these have only considered a CC analysis [3] or maximum likelihood based approaches (e.g. [17,18]) and not MI techniques. There remains a lack of evidence about the effects of missing covariate data and its handling on the performance of the survival models and no consensus on the most appropriate MI techniques to use with a survival outcome.

In addition, no definitive guidelines appear to exist on the allowable proportion of missing data to validly apply MI techniques [19]. With a single incomplete covariate or outcome, Harrell [20] suggested using imputation rather than a CC analysis with 5% missingness, although Barzi and Woodward [21] suggested that a CC analysis may still be suitable with up to 10% missingness. For MAR data, MI performed well up to 25% missingness, and adequately with 50% missingness [22]. However with more than 60% missingness, the extreme levels of uncertainty about the imputed values resulted in high standard deviations and convergence problems of the imputation procedure with MI [21]. With missing not at random (MNAR) data, where the probability of missingness is associated with the unobserved values [8], variance estimates were affected when more than 5% of the data were missing [22]. All of these findings relate to an incomplete outcome or a single covariate and not to the situation with multiple incomplete covariates, where the missingness could relate to the level of an individual covariate or to the proportion of cases that have incomplete data for at least one covariate.

This paper reports the results of an extensive simulation study that aimed to assess the effects of applying different standard approaches to handle missing data in more than one covariate when fitting a Cox proportional hazards model to the full set of covariates. This simulation study investigated how the performance of the model was affected by varying amounts of missingness and different missing data mechanisms. We aimed to determine the maximum allowable proportion of missingness to validly apply these missing data techniques.

## Methods

Details of the simulation procedures used within this simulation study are provided below. All simulations were performed using the freely available R statistical software [23], thus allowing all researchers access to any suitable methods identified.

### Generating the datasets

**Figure 1:**
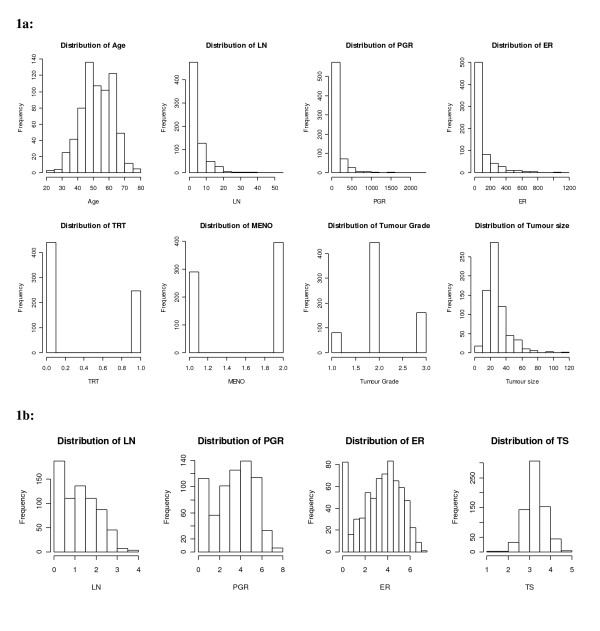
**a: Distribution of the covariates for the German breast cancer dataset; b: Distribution of the transformed continuous covariates in the German breast cancer dataset**.

To reflect reality, a German breast cancer dataset [24] formed the motivating example for generating the simulated datasets; it assessed the prognostic ability of eight covariates (Table 1) in relation to recurrence free survival. The non-normally distributed continuous covariates of lymph nodes (X_2_), progesterone receptor (PGR) level (X_3_), oestrogen receptor (ER) level (X_4_) and tumour size (X_8_) had varying degrees of skewness (2.87, 4.77, 3.07 and 1.77 respectively; Figure 1). Some covariates were highly associated, e.g. age (X_1_) and menopausal status (X_6_; *r *= 0.77) and X_3 _and X_4 _(*r *= 0.39); others were moderately correlated, e.g. X_2 _and X_8 _(*r *= 0.33) and hormonal treatment (X_5_) and X_6 _(*r *= 0.28).

**Table 1:** Data structure for the breast cancer dataset and associated means and standard deviations (SDs) after suitable transformation

Covariate	Variable Type	Groupings/Measurement	Label	*X*	Mean(SD)
**Age**	Continuous	Years	Age	*X*_1_	53.05(10.12)

**Lymph nodes**	Continuous	Number of	LN	*X*_2_	1.16(0.94)

**Progesterone receptor**	Continuous	fmol	PGR	*X*_3_	3.35(1.93)

**Oestrogen receptor**	Continuous	fmol	ER	*X*_4_	3.35(1.84)

**Hormonal treatment**	Binary	1 = Yes, 0 = No	TRT	*X*_5_	0.36(0.48)

**Menopausal status**	Binary	0 = Pre, 1 = Post	MENO	*X*_6_	0.58(0.49)

**Tumour group**	Binary	0 = Grade I, 1 = Grade II/III	TG	*X*_7_	0.88(0.32)

**Tumour size**	Continuous variable categorised	1 = ≤20 mm, 2 = 21-30 mm, 3 = >30 mm	TS	*X*_8_	3.27(0.46)

**Note: **Data from the breast cancer dataset for X_2 _and X_8 _were log transformed; X_3 _and X_4 _were transformed using log(X+1).

For simplicity, the covariate data were generated using an underlying multivariate normal distribution [25] with the covariate means and covariance matrix obtained from the German breast cancer study data after suitable transformations (Table 1, Figure 1b). A log transformation was used for the continuous covariates X_2 _and X_8 _and a log (*X *+ 1) transformation used for X_3 _and X_4 _to avoid taking logs of zero. The generated covariate data were back transformed onto their original scales, e.g. using exponential transformations, prior to any analyses being performed. A cut-point of 0.5 was used to obtain the three binary covariates (X_5_, X_6_, X_7_) and the same cut-points as in the original data were used for the categorical covariate (X_8_). Two dummy variables for X_8 _were created to indicate values of 21-30 mm or not and >30 mm or not respectively. Continuous covariates were truncated using their upper observed limits to produce realistic values and reasonable estimates for the mean and standard deviations that were not too dissimilar from the original dataset.

For each case a linear predictor was calculated as the sum of the products of the generated covariate values and the associated regression coefficient estimates obtained from fitting the full Cox proportional hazards model to the motivating dataset, such that:

All continuous covariates were assumed to have a linear effect on the log relative hazard. An uncensored survival time was generated for each case assuming an exponential distribution with a hazard rate of 0.00027, which approximated the hazard seen in the breast cancer dataset, and their associated linear predictor [26]. A censored time was also generated for each case using an exponential distribution with a hazard rate of 0.0002 to give approximately 35% censored observations. A smaller censoring rate than that seen in the breast cancer dataset ensured there were a sufficient number of events to fit a prognostic model for all levels of missingness. The required survival time was then defined for each case as the minimum of the uncensored and censored survival times and the event status determined accordingly.

A sample size of 1000 cases was used for all simulations, which represented the average sample size observed in a literature review of 100 reported prognostic factor studies [1].

### Number of simulations

The whole simulation process was repeated 1000 times, which enabled the smallest regression coefficient for X_4 _to be estimated with at least 20% accuracy and all remaining regression coefficients estimated to within 10% accuracy of their true values [25]. The true values were obtained from fitting a Cox proportional hazards model to the motivating breast cancer data. Independent random samples were generated using different starting seeds that were separated by at least the sample size [25].

### Imposing missing data mechanisms

Missingness was imposed on four covariates: X_3_, X_2_, X_5 _and X_8_. A case was said to be incomplete if they had at least one missing covariate, but each case could have up to four covariates missing. Five overall rates of missingness of 5, 10, 25, 50 and 75% per case were considered to explore the effects with small, medium and large amounts of missingness. A moderately independent simulation strategy [25] was adopted, utilising the same set of 1000 datasets each time but with different values randomly deleted through using different starting seeds. This approach strengthens the comparison between different methods as it eliminates any sampling variability leaving all methods striving for exactly the same results, whilst allowing variability to exist between simulated datasets and amounts of missingness.

Data for four incomplete covariates (performance status, albumin, grade and residual disease) from an ovarian cancer study [27] provided empirical evidence of realistic patterns and frequencies of missing data and associations between the missingness of each covariate. The amount of missingness imposed on each of the four covariates, X_3_, X_2_, X_5 _and X_8 _were approximately 70%, 55%, 20% and 10%, respectively, of the overall amount of cases with any missing data. Dependencies between the missingness indicators for the incomplete covariates were generated such that 35% of incomplete cases were missing both X_2 _and X_3_, 10% were missing X_5 _and X_2_, and 5% were missing X_8 _and X_3_.

Four multivariate missing data mechanisms were imposed, since these are the least studied mechanisms, yet the most appropriate to real life situations. The mechanisms investigated were missing completely at random (MCAR), MAR, MNAR [8] for all incomplete covariates and a combined multivariate mechanism that imposed a different mechanism on each incomplete covariate (Table 2). The MAR and combined mechanisms involved both covariate-dependent and outcome dependent mechanisms. Separate logistic regression models for each incomplete covariate were used to model the probability of the covariate being missing according to the appropriate missing data mechanism (Tables 2 and 3). A probability of missingness for each incomplete covariate was calculated for each case and compared against a random value from the Uniform [0, 1] distribution. The covariate value for a case was set to be missing if their uniformly distributed value did not exceed the calculated probability.

**Table 2:** Specification of the missing data mechanisms to be imposed

Mechanism	X_3 _(PGR)	X_2 _(LN)	X_5 _(TRT)	X_8 _(TS)
**MCAR**	β_0_	β_0 _+ ln(OR)M_X3_	β_0 _+ ln(OR)M_X2_	β_0 _+ ln(OR)M_X3_

**MAR**	ln(0.8)X_4_	ln(3)X_1_	ln(0.7)ln(t)	ln(7)X_7_

**MNAR**	ln(1.3)X_3_	ln(0.6) X_2_	ln(8)X_5_	ln(0.9)X_8_

**COMBINED**		ln(0.7)ln(t) + ln(0.3)X_5_	ln(3)X_1_	ln(0.9)X_8_

**Note: **A logistic regression model was used to model the probability of missingness for each incomplete covariate. The entries in the table represent the variables associated with the missingness of each incomplete covariate. For MAR, MNAR, and the combined mechanism, the terms given are extra to those for the MCAR mechanism, e.g. the MAR mechanism for X_2 _is

where β_0 _is the intercept, estimated by solving the above equation using the specified probabilities of missingness for X_2 _and X_3 _and the average covariate value of X_1_, M_X3 _is the missingness indicator for covariate X_3_, which equals 1 if an observation is missing and 0 if the value is observed and OR is odds ratio for the relationship between the missingness of X_2 _and X_3_, and is obtained from Table 3. The coefficients for the variable associated with the mechanism were modified from relationships with missing data seen in another study [27] to provide significant associations. All continuous variables including survival (t) were standardised by dividing by the standard deviation. When the mechanisms included other covariates subjected to missingness, the original complete data were used.

**Table 3:** Odds ratios (OR) to be specified in the missing data mechanisms given in Table 2

Mechanism	OR	Missingness (%)				
for:	for:	5	10	25	50	75
**X_2_**	**M_X3_**	101.12	45.5	15.68	5.50	2.17
**X_5_**	**M_X2_**	42.04	20.78	7.41	3.00	1.51
**X_8_**	**M_X3_**	45.14	14.23	5.44	1.92	0.92

**Note: **M_Xi _is the missingness indicator for covariate X_i_, which equals 1 if an observation is missing and 0 if the value is observed.

OR represents the odds of having two variables with missing observations, and was calculated using the proportion of missing values for each variable and the degree of overlap between variables for each of the five overall amounts of missingness to be imposed.

### Analysis and outcomes of interest

A Cox proportional hazards model including all eight covariates was fitted to each dataset. A linear relationship was assumed for all continuous covariates as used in the data generation process.

The outcomes of interest were the regression coefficients, associated standard errors (SE) and the significance of the covariates in the regression model. The average regression coefficient estimates over all simulations were assessed using the bias from the true value [12], the percentage bias and the coverage [28]. The effect of the missingness on the overall model performance was assessed using the likelihood ratio chi-square test [20], the model's predictive ability using Nagelkerke's R^2 ^statistic [20], the prognostic separation D statistic [29] and the 2-year predicted survival probability.

The bias introduced from maximising the partial likelihood estimator and not the full estimator when fitting a Cox regression model [30] in addition to any bias due to the data generation process impedes the detection of any additional bias incurred due to the missing data and its handling. Hence the average regression coefficient estimates and associated empirical SE (i.e. the standard deviation of the estimates across simulations) from performing a large simulation study with no missingness involving 20,000 replications formed the true values against which the missing data simulations were compared.

### Missing data methods

A CC analysis, single imputation (SI) using predictive mean matching [9] and five MI techniques, available within the R statistical software, were investigated (Table 4). All are suitable for imputing multivariate arbitrary missingness and are easily accessible. The MI techniques included two data augmentation approaches [31], one assuming a joint multivariate normal distribution (NORM) and one using a general location model (MIX); a regression switching approach (MICE) and the application of predictive mean matching after regression switching (MICE-PMM) [9]. The final MI approach (aregimpute) fitted separate flexible additive imputation models to each incomplete covariate [20].

**Table 4:** Summary of the missing data methods investigated

Method Label	Method Description	Library used within R statistical software	Number of iterations
**CC**	Complete case analysis: Analyses only cases with complete data for all covariates		-

**SI**	Single imputation performed using PMM	'*pmm*' function in 'mice'	20

**MI-NORM**	Multiple imputation (MI) using data augmentation approach [31] with a multivariate normal assumption for all variables	'norm' [41]	100

**MI-MIX**	MI using data augmentation approach using a general location model	'mix' [42]	100

**MI-MIX-no truncating**	MI using data augmentation approach using a general location model, but imputed values are not truncated to within plausible range	'mix' [42]	100

**MI-MICE**	MI using regression switching imputation [9]. Linear model are used for continuous covariates and logistic model for binary covariates and dummy variables for categorical covariates	'mice' [43]	20

**MI-MICE-PMM**	MI using MICE with PMM	'*pmm*' function in 'mice' [43]	20

**MI-MICE-PMM-no transformation**	MI using MICE with PMM without transforming the incomplete covariates	'*pmm*' function in 'mice' [43]	20

**MI-Aregimpute**	MI using flexible additive imputation models [20] with PMM	'*aregImpute*' function in 'Hmisc' [44]	1

**Key: **PMM = predictive mean matching; MI = multiple imputation

For all imputation approaches, the imputation model included all eight covariates in addition to the survival time and event status, indicating whether a case had the event or was censored at the time of analysis [9]. A logarithmic transformation was used for survival time and the incomplete continuous covariates to make the assumption of normality more applicable [9]. All imputed values were rounded to plausible values, where necessary. Twenty imputations were performed for each MI approach to provide a relative efficiency of at least 96% [7] compared to having an infinite number of imputations for the five amounts of missingness to be imposed from 5% to 75%.

#### Combining estimates of the outcomes of interest after MI and over all simulations

Estimates of the outcomes of interest after MI were combined following proposed guidelines [32]. Rubin's Rules [7] were used to combine each of the regression coefficient estimates, the prognostic separation D statistic and the predicted survival estimates after a complementary log-log transformation. An overall MI p-value from the Wald test for assessing the significance of each covariate in the regression model was also determined using Rubin's Rules [7]. An overall significance estimate for the likelihood ratio statistic was obtained using the method for combining *X*^2 ^statistics [33]. The median and inter-quartile ranges of the *m *Nagelkerke's R^2 ^statistics [32] were calculated for each of the simulated datasets. Any deficiencies in the model performance measures and approaches for combining these estimates after MI should be similar across missing data methods and therefore still allow a valid and worthwhile comparison. After performing all 1000 simulations, the outcomes of interest were summarised, in general using the average value over all simulations or using the median value, where appropriate.

## Results

The results from performing MI using MICE, NORM and MIX were indistinguishable for all mechanisms and therefore only the results using MICE are presented. Firstly, the results from imposing a multivariate MAR mechanism are reported for all missing data methods.

### Results from imposing a multivariate MAR mechanism

#### Regression coefficient estimates from the Cox proportional hazards model

**Figure 2:**
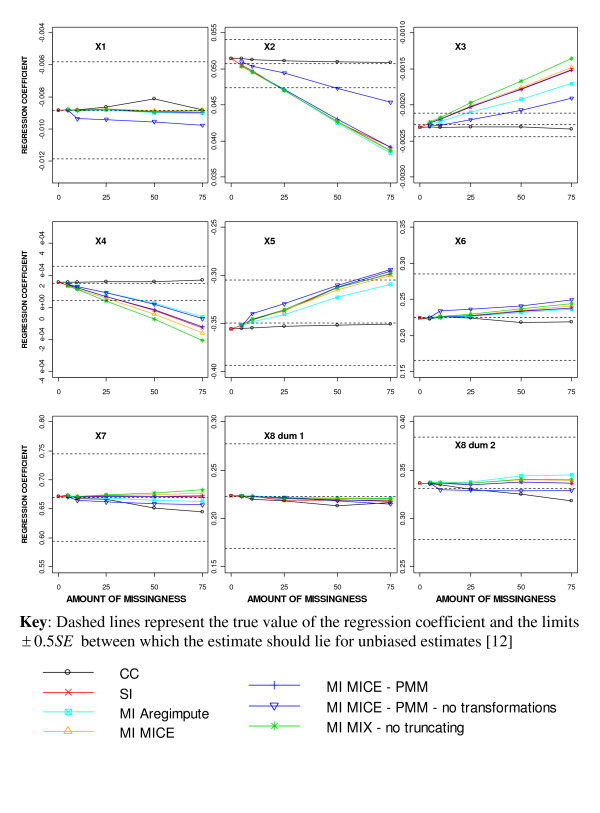
**Regression coefficient estimates for different missing data methods for increasing percentage of MAR missingness**.

The regression coefficient estimates obtained from performing different missing data methods with increasing amounts of MAR missingness are shown in Figure 2. The regression coefficient estimates after performing a CC analysis remained within the limits for unproblematic estimates [12] of ± 0.5SE for all levels of missingness and were generally closer to the true value for all covariates than after using SI or MI (Figure 2). For SI and most MI approaches, the regression coefficient estimates were more than 0.5SE away from the true value for the two incomplete continuous covariates (X_2 _and X_3_) and X_4_, the covariate highly correlated with X_3_, when 25% or more of the cases had at least one covariate missing. For X_2 _and X_3_, the percentage bias exceeded the allowable 10% with 50% missingness using MICE-PMM without transformations but with only 25% missingness for all other MI approaches. The regression coefficient estimate for X_4 _was extremely close to zero and therefore the percentage bias was not meaningful for this covariate. The percentage bias remained within 10% for the remaining five covariates, except for X_5 _with 50% missingness using all MI approaches and for X_1 _and X_6 _with 75% missingness using MICE-PMM without transformations.

For the highly skewed continuous covariates X_2_, X_3 _and X_4_, the least biased regression coefficient estimates were produced when MI was performed using MICE-PMM without transformations. In contrast, more bias was seen for the regression estimates for X_1_, X_5_, and X_6 _using this approach. When the imputed values were not truncated to within a plausible range (MIX-no truncating), all regression coefficient estimates tended to be slightly more extreme than with all other MI approaches.

#### SE of the regression coefficient estimates

**Figure 3:**
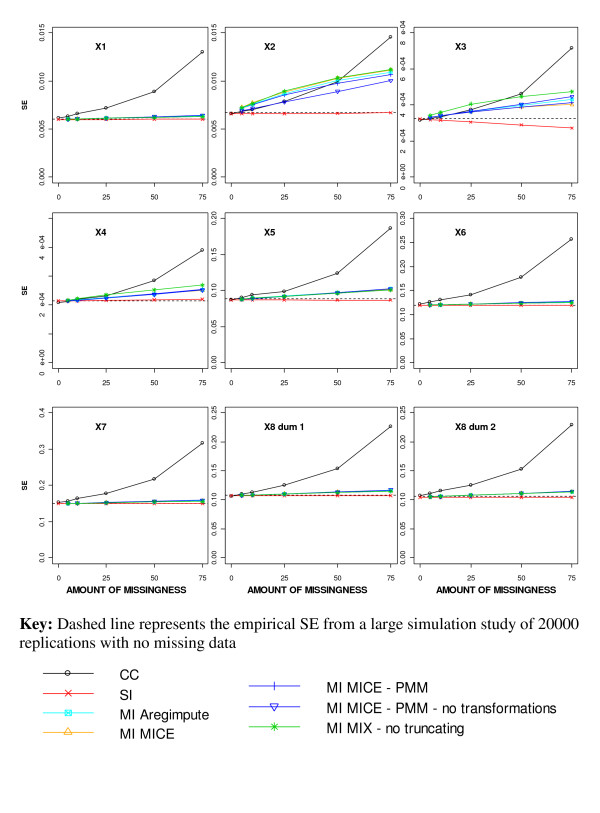
Average standard error (SE) estimates for different missing data methods for increasing percentage of MAR missingness

The average SE estimates for the incomplete covariates from all MI approaches were similar and fell between the estimates from CC analysis and those obtained after SI (Figure 3). Applying SI or MI did not affect the average SE for the complete covariates, but the estimates after performing a CC analysis were considerably increased, reflecting the decrease in sample size analysed.

#### Coverage of the true value

**Figure 4:**
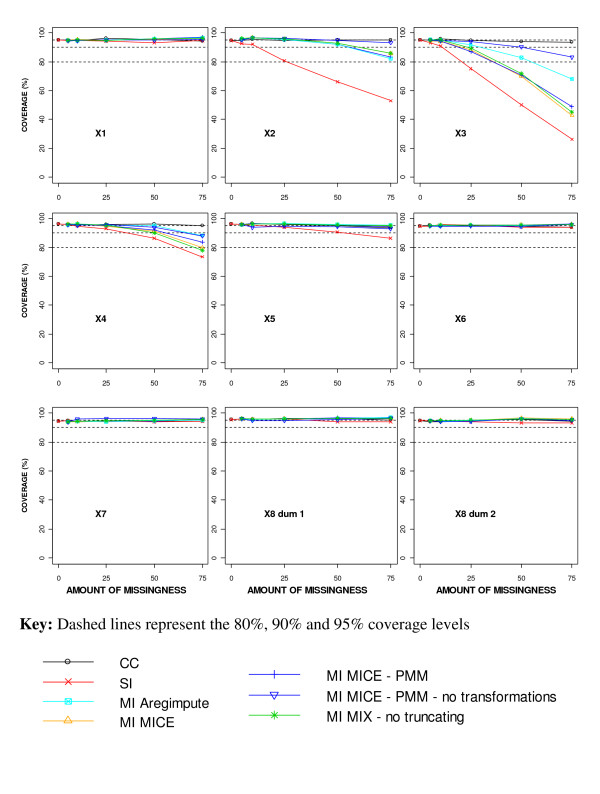
**Coverage of the regression coefficient estimates for different missing data methods for increasing percentage of MAR missingness**.

Figure 4 shows the coverage of the regression coefficient estimates for the different missing data methods in relation to an increasing percentage of MAR missingness. The coverage of the true value within the confidence limits constructed after performing a CC analysis remained around the nominal 95% level for all amounts of missingness (Figure 4). The underestimated SE with SI resulted in much shorter confidence intervals than with the other missing data methods and poorer coverage especially for the incomplete covariates and also X_4_. Even with 10% missingness, the coverage for the regression coefficient estimates associated with X_2 _and X_3 _was around 90% using SI.

The coverage using the different MI approaches remained around the nominal 95% level irrespective of the amount of missingness for all covariates except the highly skewed covariates of X_2_, X_3 _and X_4 _(Figure 4). The coverage for X_2 _and X_4 _fell below 90% with 75% missingness for all MI approaches, except using MICE-PMM without transformations for X_2_, which still had coverage of 93% with 75% missingness. The coverage for X_3_, the covariate with a highly skewed distribution and the most missingness imposed, fell below 90% with 50% missingness using MICE-PMM without transformations and the *'aregImpute' *function, but fell below 90% with only 25% missingness for all other MI approaches.

#### Significance of covariates in the prognostic model

**Figure 5:**
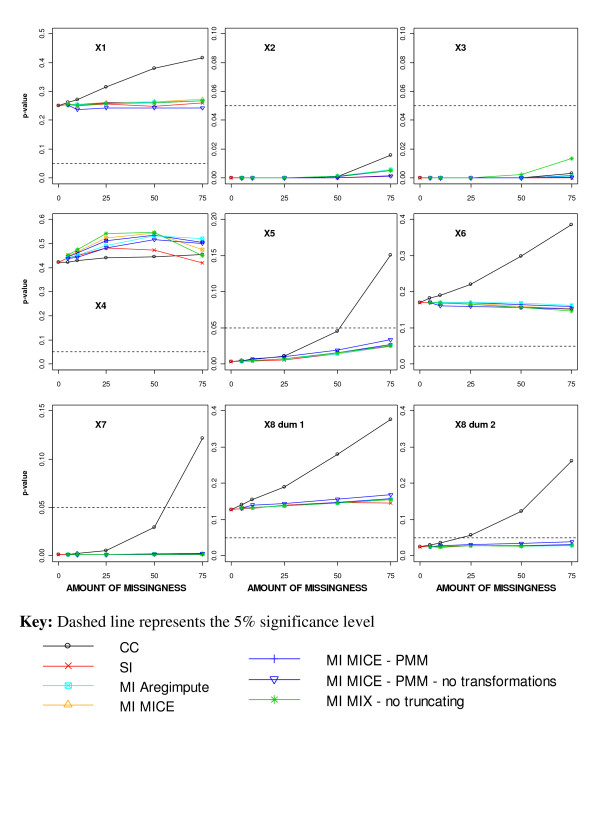
**Significance of the covariates in the prognostic model for different missing data methods and increasing percentage of MAR missingness**.

The significance of the covariates in the prognostic model after applying different missing data methods to increasing percentage of MAR missingness is displayed in Figure 5. After performing a CC analysis, all covariates in the model became less significant irrespective of their completeness, due to the reduction in sample size. The borderline X_8 _dummy variable representing group 3 (>30 mm) became non-significant at the 5% level with 25% or more missingness, and the covariates X_5 _and X_7 _became non-significant with 50% or more missingness (Figure 5).

With MI and SI, none of the covariates changed their significance in the model at the 5% level (Figure 5). However, the binary covariate X_5 _and the dummy variable for X_8 _representing group 3 (>30 mm) became borderline significant with increasing amounts of missingness.

#### Model performance measures

**Figure 6:**
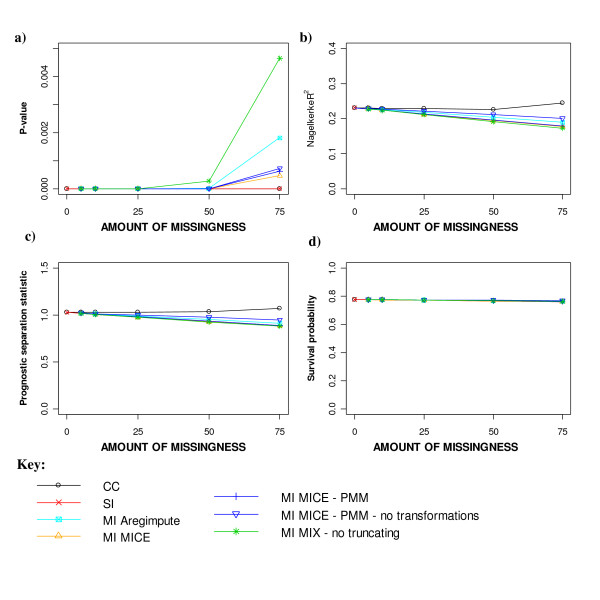
**Model performance measures for different missing data methods for increasing percentage of MAR missingness**. a) Likelihood ratio test, b) Nagelkerke R2 statistic, c) Prognostic separation D statistic and d) Predicted 2-year survival from Cox model.

Figure 6 provides the estimates of the model performance measures for the different missing data methods applied to increasing levels of MAR missingness. The likelihood ratio statistic remained highly significant for all missing data methods and levels of missingness (Figure 6a). However, discrepancies appeared between the MI approaches with 50% missingness. Of the MI procedures, applying MICE had the least effect on the significance of the likelihood ratio statistic, whereas MIX without truncating to plausible values reduced the significance of the model the most. Estimates of both the Nagelkerke's R^2 ^statistic and the prognostic D statistic tended to worsen similarly for SI and all MI approaches as the amount of overall missingness increased, although slightly better R^2 ^estimates were seen with MICE-PMM without transformation and the *'aregImpute' *function (Figure 6b and 6c). The overall predicted survival probabilities at 2 years were relatively unaffected by the methods used to handle the MAR data or the amount of missingness imposed (Figure 6d).

### Results from imposing other missingness mechanisms

No apparent differences from the above results for a multivariate MAR mechanism were seen in the results after imposing a multivariate MCAR or combined missing data mechanism. The similarity of results for the multivariate MAR mechanism and the combined mechanism may have occurred because the MNAR mechanism was imposed on the covariate with the smallest amount of missingness and hence this mechanism had the least effect on the overall results.

**Figure 7:**
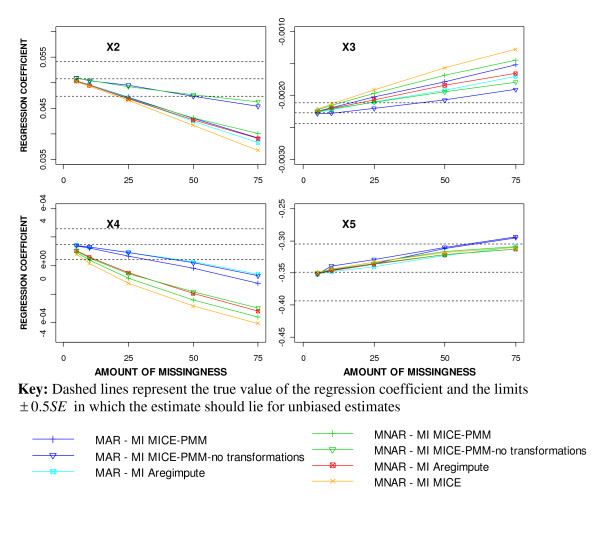
**Comparison of the regression coefficient estimates for the different MI methods after imposing MAR and MNAR mechanisms**.

**Figure 8:**
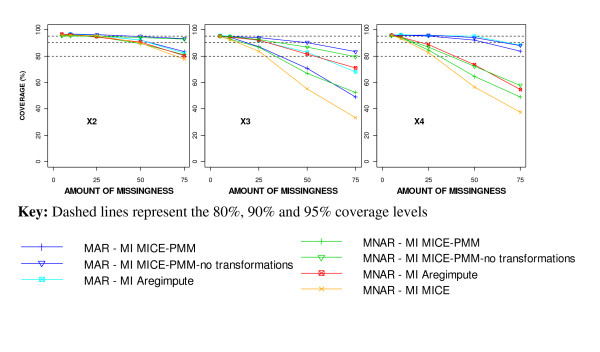
**Comparison of coverage estimates for the different MI methods after imposing MAR and MNAR mechanisms**.

The results from imposing a multivariate MNAR missing data mechanism, however, showed some discrepancies from those seen with a MAR mechanism. The regression coefficient estimates for X_3 _and X_4_, were further from the true value when a MNAR mechanism was imposed than with a MAR mechanism, but estimates for X_2 _and X_5 _were slightly closer (Figure 7). The regression coefficient estimates for X_3 _and X_4 _were more than 0.5SE away from the true value [12] with 25% or more MNAR missingness using any MI approach, but only with 50% or more MAR missingness using MICE-PMM without transformations. The coverage for X_2_, X_3 _and X_4 _was worse after MI using a MNAR mechanism than a MAR mechanism (Figure 8).

## Discussion

Using a real dataset to provide a suitable structure for simulating the datasets, as in this study, simplifies the data generation procedures, avoids arbitrary choices and can aid the generalisability of the results. The simulated data were not an exact replica of the original, but provided sufficiently strong similarities to the original data to not warrant using more complicated semi-continuous distributions for PGR level (X_3_), and ER level (X_4_). Approximating the patterns of missingness seen in an incomplete dataset provided a realistic framework for simulating the missing data. The levels of missingness imposed reflected those seen in a review of prognostic modelling studies [1]. The effects of using MI when fitting prognostic models were unknown. Therefore this simulation study restricted the modelling process to including all covariates in the prognostic model and assuming linear relationships for all continuous covariates, both in the generation of the survival times and in the fitting of the prognostic model. Extensions of this research could include investigating the possible approaches for performing variable selection and fitting fractional polynomials after MI (e.g. [34]).

In this simulation study, performing a CC analysis with any multivariate missingness produced less biased regression coefficient estimates with better coverage rates than using SI or MI. However, this benefit was at the expense of larger standard errors and hence loss of efficiency due to the reduction in the sample being analysed [8]. This loss in efficiency affected the significance of the covariates in the prognostic model of the marginal prognostic covariates, making them appear non-significant with 25% or more missingness when in fact they were prognostically important. It is advisable to use a CC analysis only when fitting a Cox proportional hazards model with a reasonably small amount of missing data. Previous evidence [2,3] suggested that imposing univariate MAR missingness associated with outcome would result in biased regression coefficient estimates when using CC analysis. Demissie et al [3] found large biases when the MAR mechanisms were associated with longer survival times or event status and the covariates had large hazard ratios for survival, but not with a hazard ratio of 1 or when the missingness was associated with shorter survival times. Relatively unbiased results were found in our simulations with multivariate MAR missingness where the missingness of X_5_, the binary covariate with 20% of the total missingness, was associated with shorter survival times and the hazard ratio for X_5 _in the prognostic model was 0.7. Biased regression estimates may be more evident with more missing covariate data that is highly dependent on the outcome, especially longer survival times and event status, and with large hazard ratios.

With multivariate missing covariate data, using SI is not recommended with more than 10% missingness, due to its underestimation of the variability and corresponding detrimental effect on the coverage rates.

The results from performing simulations based on the German breast cancer dataset showed some bias, especially with over 25% missingness, for all mechanisms and MI approaches. The average SEs for all MI approaches and mechanisms were similar. They fell between the inflated estimate from the CC analysis and the underestimated SE from SI, as expected from previous research [8,35]. The coverage was unaffected and remained around the nominal 95% level for all mechanisms and covariates, except for the skewed covariates. Tang et al [36] also found that the coverage may be poorer after MI for highly skewed data. Better coverage rates were seen using MICE-PMM without transformations or the '*aregImpute*' function than with the other MI approaches and also when a MAR mechanism was imposed rather than a MNAR mechanism.

Researchers have suggested that MI approaches are fairly robust to departures from normality due to the separation of the imputation and analysis phases [12,13,37]. Any deficiencies in the assumptions and implementation of the imputation model will only affect the incomplete component of the dataset and not the whole sample [38]. Having skewed continuous data and an outcome of survival time as in this simulation study may have affected the performance of the methods under investigation, especially those which assumed an underlying normal distribution for the continuous covariates, e.g. NORM, MIX and MICE. This study highlighted the problems that can exist when the imputation and analysis models differ and the model assumptions may not be fully satisfied. The bias seen in this simulation study even when the MAR mechanism assumption was correct may be an artefact of the transformations used in the imputation process [39]. Not only are the incomplete covariates transformed for imputation and then back-transformed prior to analysis, but the survival times are also transformed in the imputation model and then fitted using an alternative model. Imputing without transformation can reduce the bias in the mean estimate but distort other aspects of the distributional shape [39]. Log transformations were used for the continuous covariates in the data generation process. However, as the simulated data were then truncated to resemble the real data, applying the same transformations in the imputation process failed to satisfy normality. No other simple power transformations sufficiently improved normality or provided more plausible imputations.

The inclusion in the imputation model of survival time after log transformation and event status may not be the optimal choice to account for the censoring process and thus may have also introduced bias into the results. Using the Nelson-Aalen estimate of the cumulative hazard of the survival time may be more appropriate in future [40]. If the hazard rates for the survival and censoring times differ then it may be sensible to consider these times separately in the imputation model.

The MI procedures using MICE-PMM or the *'aregImpute' *function, which rely on the distributional assumptions only to match complete and incomplete responders, performed better for all missingness mechanisms than the other MI approaches. This confirmed the results from Faris et al [15] that with incomplete skewed data, MICE-PMM would be preferred to other MI Markov Chain Monte Carlo type approaches. However, caution is needed when using the *'aregImpute' *function, especially when the missingness is highly related to survival, as although the estimates for the incomplete covariates may exhibit little bias, the estimates for other prognostically important covariates may display more bias than seen with other MI approaches. Both MICE-PMM and the *'aregImpute' *function identify suitable matches from the observed data and therefore additional caution is required with small samples and with covariates with rare events as there may be a limited number of available cases to be selected as imputed values. With skewed data, values of a few cases have a lot of leverage that may distort the imputations and influence the results. Therefore it is essential to examine the distributions of the covariates requiring imputation to determine whether transformations are likely to provide reasonable estimates for the data to be analysed. With MICE-PMM, transforming the continuous covariates produced worse estimates than simply using the covariate values on their original skewed scale. Our findings suggest that if suitable transformations do not improve normality it is better to use MICE-PMM without transformations. With a fully observed normally distributed outcome and more normally distributed incomplete covariates and hence compatible imputation and analysis models, MICE-PMM may not remain the best MI approach.

In this simulation study, truncating imputed values for the continuous covariates to within the plausible range produced less bias than allowing implausible values. Schafer [31] suggested rounding values for the incomplete binary covariate to the observed values. In these simulations, where only 20% of the total missingness was imposed on the binary covariate, that approach did not produce any more bias than using the correct distributional assumption, e.g. fitting logistic regression models. Biases may be more apparent when the binary covariate has an uneven split or greater missingness [39].

From this simulation study, with incomplete skewed data, MI using MICE-PMM without transformations produced precise unproblematic estimates [12] within the allowable 10% accuracy with up to 25% missingness, but would not be recommended with 50% or more missingness for any missing data mechanism. Furthermore, with a MNAR mechanism, MI performed poorly with 25% or more overall missingness. Including variables in the imputation model that help to explain the missingness or are highly associated with the incomplete covariates themselves, can reduce the effect of an MNAR missing data mechanism [8]. With less enriched imputation models, and datasets where there is little correlation between variables, the results from the MNAR may be even more extreme than seen here. Further research is required to assess whether alternative MI procedures or fully Bayesian approaches that can model the skewness of the covariate distribution and the missing data mechanism may be more appropriate when there is more than 25% missingness.

The true performance of the various missing data methods is likely to vary in relation to the underlying distribution of the covariates, the correlations between these variables as well as with different missing data mechanisms and associations between the outcome and the covariates with missing data. Therefore, the generalisability of the results from this simulation study, however rigorous, is limited due to reflecting the data from a single real prognostic study and imposing a restricted number of missing data mechanisms. Confirmatory investigations are required to examine the extent to which these findings are consistent across alternative populations, distributions and clinical contexts.

## Conclusions

For approximately 10% or less missingness, it remains unclear whether the benefits of MI, including efficiency and utilising all data, outweigh the simplicity of a CC analysis. With increasing amounts of missingness, the benefits of MI over a CC analysis become clearer. When some data are skewed, as in this simulation study, MICE-PMM may be the preferred MI approach provided that less than 50% of the cases have missing data and the missing data are not MNAR.

## Competing interests

The authors declare that they have no competing interests.

## Authors' contributions

AM participated in the design, coordination and analysis of this study and drafted the manuscript. DGA participated in the design of the study, the interpretation of the data and helped in the writing of the manuscript. PR participated in the design of the study, the interpretation of the data and revision of the manuscript. RH advised on the design and interpretation of the study, and participated in the revision of the manuscript. All authors have read and approved the final manuscript.

## Pre-publication history

The pre-publication history for this paper can be accessed here:

http://www.biomedcentral.com/1471-2288/10/7/prepub
